# Effect of Molecular Weight on Phase Equilibrium in the Polystyrene–Poly(methyl methacrylate) System

**DOI:** 10.3390/molecules28135259

**Published:** 2023-07-07

**Authors:** Anatoly E. Chalykh, Uliana V. Nikulova

**Affiliations:** Frumkin Institute of Physical Chemistry and Electrochemistry Russian Academy of Sciences (IPCE RAS), 31, Bld. 4 Leninsky Prospect, Moscow 119071, Russia; ulianan@rambler.ru

**Keywords:** phase state diagram, mixing thermodynamics, simulation, polystyrene, poly(methyl methacrylate), UCST, solubility, interaction parameter, FTIR

## Abstract

Data on the solubility of oligomer polystyrene (PS) and poly(methyl methacrylate) (PMMA) of various molecular weights have been obtained. The binodal and spinodal curves of the phase state diagram with the upper critical solution temperature (UCST) are constructed through simulation within the framework of the Flory–Huggins theory. The influence of the molecular weight of polymers on the contribution to their mixing has been compared, and correlation curves have been plotted. The interaction parameters were calculated and the mixing thermodynamics of the components was evaluated. The largest contribution was made by the entropy component. Also, it has been shown using IR spectroscopy that there is no interaction between the functional groups of polystyrene and poly(methyl methacrylate) in a homogeneous mixture.

## 1. Introduction

In the thermodynamics of mixing polymers, the problem of phase equilibria of polymer systems and the influence of the molecular masses of components on them is of particular importance [1,2,3,4]. Information about the phase diagram of polymer–polymer systems ultimately determines the phase and supramolecular structure of composite materials, the conditions for their synthesis and processing, as well as the thermodynamic stability of materials during their operation and storage [5,6].

High-molecular-weight polymers often practically do not mix with each other. But, when one of the components is an oligomer, their solubility in each other is greatly improved. Promising materials from the point of view of fundamental studies of solubility, the construction of phase diagrams, and analysis of the thermodynamics of mixing polymer–polymer and oligomer–polymer systems are compositions of polystyrene (PS) and poly(methyl methacrylate) (PMMA). First, at present, monodisperse characterized fractions of these polymers have been developed and commercially produced in a wide range of molecular weights (from 1 kDa to 1000 kDa). Second, methods for measuring the translational mobility of macromolecules [7] have been developed; using these systems as an example, the sizes of coils of individual macromolecules have been determined [8], and their coefficients of mutual and self-diffusion have been estimated [9]. Finally, the temperature conditions and the mechanisms of the thermal decomposition of polymers have been identified for these systems [10].

Previously, solubility studies in PS–PMMA mixtures were also carried out using cloud point methods, and phase diagrams were constructed or modeled within the frameworks of various theories [11,12,13,14]. This has been carried out only for polymers with molecular weights of up to 13 kDa. But, in industry, 100–300 kDa polymers are used. Blends of these homopolymers are often investigated for the possible improvement of some properties of individual components (for example, an increase in the glass transition temperature of PMMA [15]). Also, the PS–PMMA system is often a model object for fundamental research on morphological evolution in thin films [16]. It is important to understand the structure that will be formed when oligomer PS of various weights is introduced into PMMA. In the synthesis of copolymers of styrene and methyl methacrylate, the total molecular weight can also reach large values, and the dimensions of one block can vary over a wide range. How will the interaction, for example, of individual blocks with each other be carried out in this case? Will they tend to form a homogeneous mixture or form separate phases and create stress on chemical bonds between blocks at the phase boundary? Copolymers of styrene and methyl methacrylate are widely used, and here it is important to understand not only the most optimal conditions for their synthesis, but also the relationship between the obtained structure and the required properties [17,18]. But, to understand this relationship, an accurate comprehension of the thermodynamics of mixing homopolymers of different molecular weights is necessary. Understanding the processes of interaction between oligomer PS and oligomer PMMA or oligomer PS and polymer PMMA makes it possible to more accurately represent and predict the properties of not only their mixtures, but also the structure and properties of copolymers based on them. All this forms the basis for interpreting the solubility and interaction between the components of the system in the study of the phase equilibria of a ternary systems PS–PMMA–copolymer of styrene and methyl methacrylate [19,20].

That is why the goal of this work was to carry out correct and systematic experimental studies investigating the influence of the molecular weight of PS and PMMA homopolymers on their phase equilibria, compatibility, and thermodynamic mixing parameters in a wide range of temperatures, molecular weights, and compositions.

## 2. Results and Discussion

**Figure 1:**
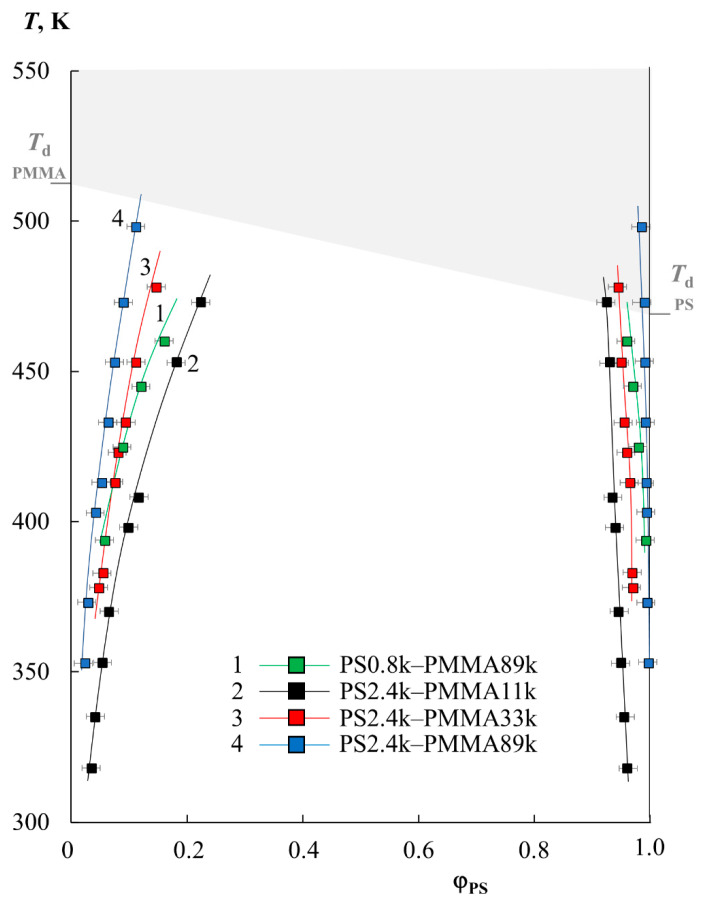
The binodal curve fragments obtained via optical interferometry for the PS0.8k–PMMA89k (1, green squares), PS2.4k–PMMA11k (2, black squares), PS2.4k–PMMA33k (3, red squares), and PS2.4k–PMMA89k (4, blue squares) systems. The gray zone corresponds to the area of thermal degradation.

Figure 1 shows the data on the solubility of PS and PMMA of various molecular weights (PS0.8k–PMMA89k, PS2.4k–PMMA11k, PS2.4k–PMMA33k, PS2.4k–PMMA89k), obtained under isothermal conditions for a temperature range limited by the glass transition temperatures of polymers (bottom) and degradation temperatures (top). It should be especially noted that the difference in the refractive indices of the studied polymers was approximately Δ*n* = 0.07–0.09, which made it possible to form about 25–30 interference fringes in the interdiffusion zone with a concentration increment per fringe of the order of 0.25–0.3 volume fractions. Based on this, we were able to observe the entire concentration range at once with a step of 2.5–3%. It has been established that the oligomer blend PS0.8k–PMMA2k is characterized by a complete compatibility over the entire temperature range. Such behavior is often expected for oligomer components; for example, for the polystyrene–polyethylene system of similar molecular weights, we obtained data on limited solubility in the range of 390–410 K and constructed a phase diagram with UCST [21]. Systems such as epoxy oligomers with nitrile butadiene rubber behave similarly [22].

It is clearly seen (Figure 1) that the solubility of PS and PMMA decreases with an increasing molecular weight of the components. For example, when going from PS0.8k to PS2.4k, their solubility in the PMMA89k matrix at 400 K decreases from 6 to 4%. And, for PS2.4k at the same temperature, the solubility in PMMA decreases from 10 to 4% with an increase in the PMMA molecular weight from 11 to 89 kDa. In this case, the phase state diagram is not symmetrical; the left branch of the binodal is located in the region from 2 to 25% PS in the mixture and shows a high solubility, while the right branch is shifted to the region of dilute solutions relative to PMMA, and the solubility of PMMA in PS tends to zero. With an increase in temperature, for all the studied systems, the mutual solubility of the components increases, which suggests the presence of UCST. However, fragments of the obtained binodal curves in this temperature range reach the zone of the thermal degradation of polymers (gray zone), which makes it difficult to experimentally determine the parameters of the critical point and estimate the position of the binodal curves’ dome and the two-phase state regions at elevated temperatures. Nevertheless, we tried to estimate the position of the binodal dome because it is at such temperatures that the industrial processing of polymer mixtures can take place.

Previously, we proposed a new approach to simulation phase diagrams from fragments of experimental binodal curves [23,24], which is based on the Flory–Huggins theory and involves the extrapolation of the temperature dependence of the interaction parameter χ. In accordance with this approach (detailed in supplementary materials), using the experimental data on the compositions of coexisting phases (φ) at different temperatures and the values of the degrees of polymerization (r), the interaction parameters χ were calculated using Equation (1), and the numerical values of the interaction parameter ($\chi_{\mathrm{cr}}$) and concentration at the critical point ($\varphi_{1,\mathrm{cr}}$) were estimated using Equations (2) and (3), respectively (Table 1). The error in calculating these quantities directly depends on the error in determining the compositions of coexisting phases. Accordingly, if for φ it is 2.5–3%, then for χ it is 5–6%. At the same time, three significant digits were retained for the numerical values of the calculated values.

(1) $$\chi =\frac{\frac{\ln\left(\varphi_{1}^{\prime \prime }/\varphi_{1}^{\prime }\right)}{r_{1}}-\frac{\ln\left(\varphi_{2}^{\prime \prime }/\varphi_{2}^{\prime }\right)}{r_{2}}}{2\left(\varphi_{2}^{\prime }-\varphi_{2}^{\prime \prime }\right)}$$

(2) $$\chi_{\mathrm{cr}}=\frac{1}{2}{\left(\frac{1}{\sqrt{r_{1}}}+\frac{1}{\sqrt{r_{2}}}\right)}^{2}$$

(3) $$\varphi_{1,\;\mathrm{cr}}=\frac{\sqrt{r_{2}}}{\sqrt{r_{1}}+\sqrt{r_{2}}}$$

where $\varphi_{1}$ and $\varphi_{2}$ are the volume fractions of the first (PS) and second (PMMA) components in the system, ′ and ″ refer to different coexisting phases, $r_{1}$ and $r_{2}$ are their degrees of polymerization, and χ is the average Flory–Huggins parameter that evaluates the interaction between the components.

**Figure 2:**
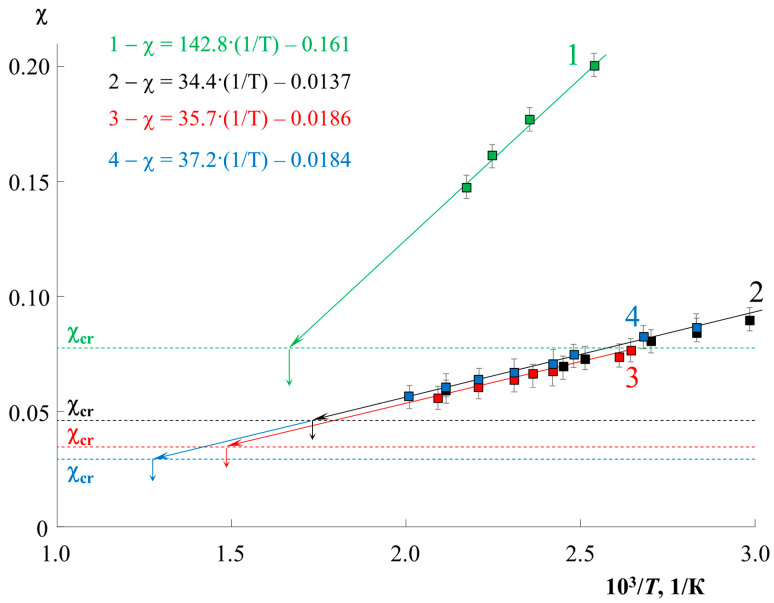
Temperature dependences of the interaction parameter for PS0.8k–PMMA89k (1, green), PS2.4k–PMMA11k (2, black), PS2.4k–PMMA33k (3, red), PS2.4k–PMMA89k (4, blue) systems, and their extrapolation to the corresponding values $\chi_{\mathrm{cr}}$ (dotted line). The interpolation equations for each system by the least squares method are presented in a similar color.

Figure 2 shows the temperature dependences of the parameter χ for all systems studied (solid lines), as well as the value of $\chi_{\mathrm{cr}}$ (dashed lines). It can be seen that the values of $\chi_{\mathrm{cr}}$, in accordance with theoretical assumptions, decrease with an increase in the molecular weight of the components. The values of the interaction parameter χ for all systems show a linear dependence for the coordinates χ−1T, and a directly proportional dependence for similar coordinates indicates a system with a UCST. The localization of the straight lines differs greatly for the *M*_PS_; for PS0.8k it is located noticeably higher and has a larger slope than for PS2.4k. At the same time, a change in *M*_PMMA_ from 11 to 89 kDa has practically no effect on the position of the straight lines. The least squares interpolation made it possible to obtain a mathematical expression for these temperature dependences in the format $\chi =A+B\cdot \frac{1}{T}$, where A and B are some numerical values (Figure 2 and Table 1). The value of the approximation reliability R^2^ (Table 1) evaluates the accuracy of the adequacy of the linear dependence. For all systems, it is in the range from 0.95 to 0.99, which indicates a high approximation accuracy. The extrapolation of these dependencies to the values of $\chi_{\mathrm{cr}}$ made it possible to obtain approximate calculated values of UCST for each system. The exact value of the UCST (Table 1) was estimated from the convergence of the binodal curves during the simulation in the resulting range.

**Figure 3:**
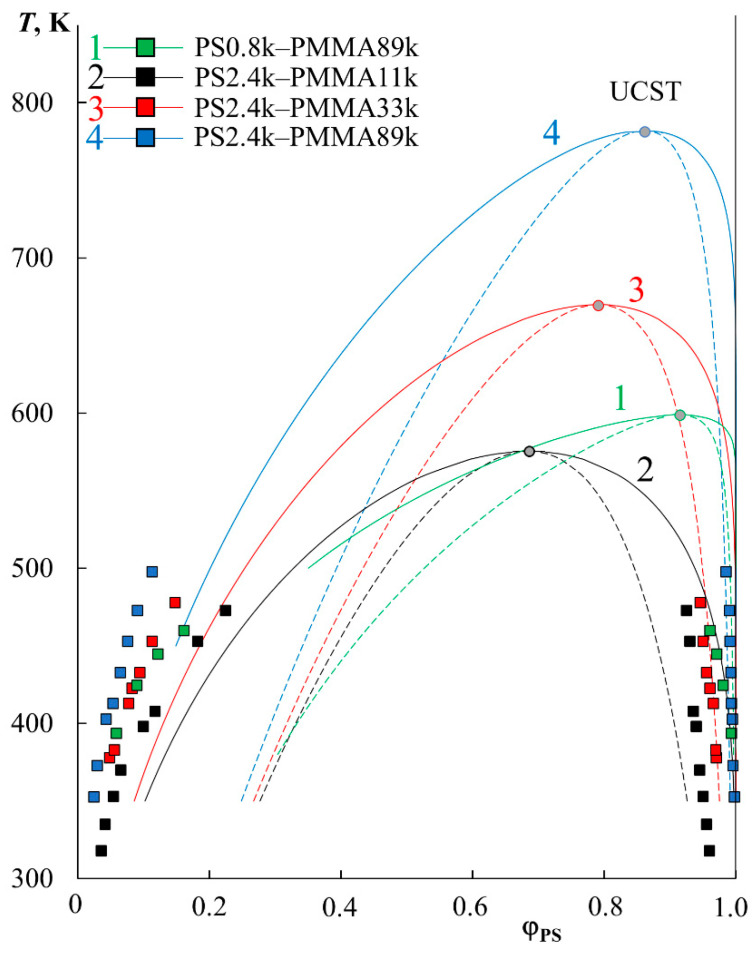
Simulation of binodal (solid lines) and spinodal curves (dotted lines) within the Flory–Huggins theory for PS0.8k–PMMA89k (1, green), PS2.4k–PMMA11k (2, black), PS2.4k–PMMA33k (3, red), and PS2.4k–PMMA89k (4, blue) systems.

The extrapolation of χ to a larger temperature range in the framework of the proposed approach [24], with calculations by the equations for the spinodal and binodal curves in the framework of the Flory–Huggins theory, made it possible to construct complete binodal (solid lines) and spinodal curves (dashed lines) for PS–PMMA systems (Figure 3). It can be seen that the phase diagrams obtained are characterized by UCST in the temperature range from 550 to 800 K. Above the binodal curves, we obtain regions of homogeneous solutions; inside the spinodal curve there are heterophase regions, and between the binodal and spinodal curves there are fields of the metastable state. The correlation with experimental data (indicated by square dots in Figure 3) is quite good, especially with the right branch of the diagram. As for the left branch of the binodal curves, the correlation between the calculated binodal curves and the experimental points is satisfactory, but not ideal. It seems to us that this is due to the error of extrapolation to a very remote temperature range. It can be seen that the correlation of the black dots (PS2.4k–PMMA11k) in Figure 3 is better than that of the blue ones (PS2.4k–PMMA89k). At the same time, the extrapolation was carried out for black dots by about 100 K, and for blue dots by almost 300 K.

**Figure 4:**
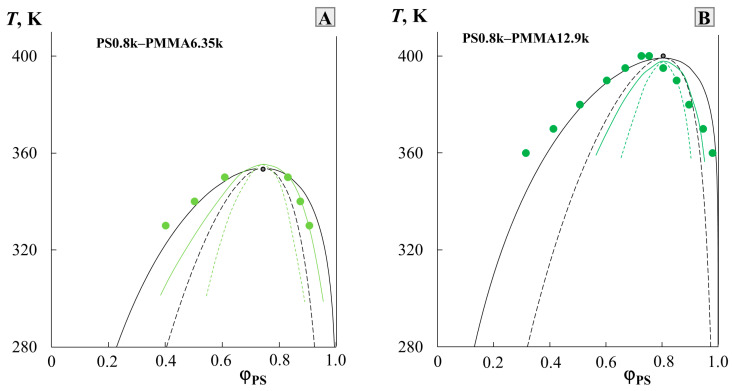
Simulation of binodal (black solid lines) and spinodal curves (black dotted lines) for PS0.8k–PMMA6.35k (**A**) and PS0.8k–PMMA12.9k (**B**) systems given in [11]. Black lines according to our methodology, and green lines correspond to the authors’ calculations for their experimental data (green dots) [11]. See detail in the text.

To test the adequacy of the prediction technique used, published numerical data on solubility in the PS0.8k-PMMA6.35k and PS0.8k-PMMA12.9k systems, obtained by the cloud point method by Ougizawa et al. [11], were selected and rearranged in other coordinates (Figure 4). They contained experimental data on complete diagrams and critical points. Also, the solid green and dotted green lines indicated the result of modeling the phase diagram in these systems based on the equation of state theory, performed by the authors in the same article. According to our methodology, data on the compositions of coexisting phases were used to construct the temperature dependence of χ, to determine the linear equation describing this dependence, to determine the parameters of the critical point (the data are presented in Table 1), and to simulate the binodal and spinodal curves (shown in solid black and dotted black lines, respectively, in Figure 4). It is clearly seen that in contrast to the calculation within the framework of the equation of state theory used by the authors of [11], the phase diagrams constructed by us perfectly correlate with the experimental data on solubility.

Solubility data and binodal fragments in PS–PMMA systems were processed similarly by a number of other authors [12,13,14], who mainly used the average molecular weights of polymers. The calculated results of all characteristics for these systems are presented in Table 1.

**Figure 5:**
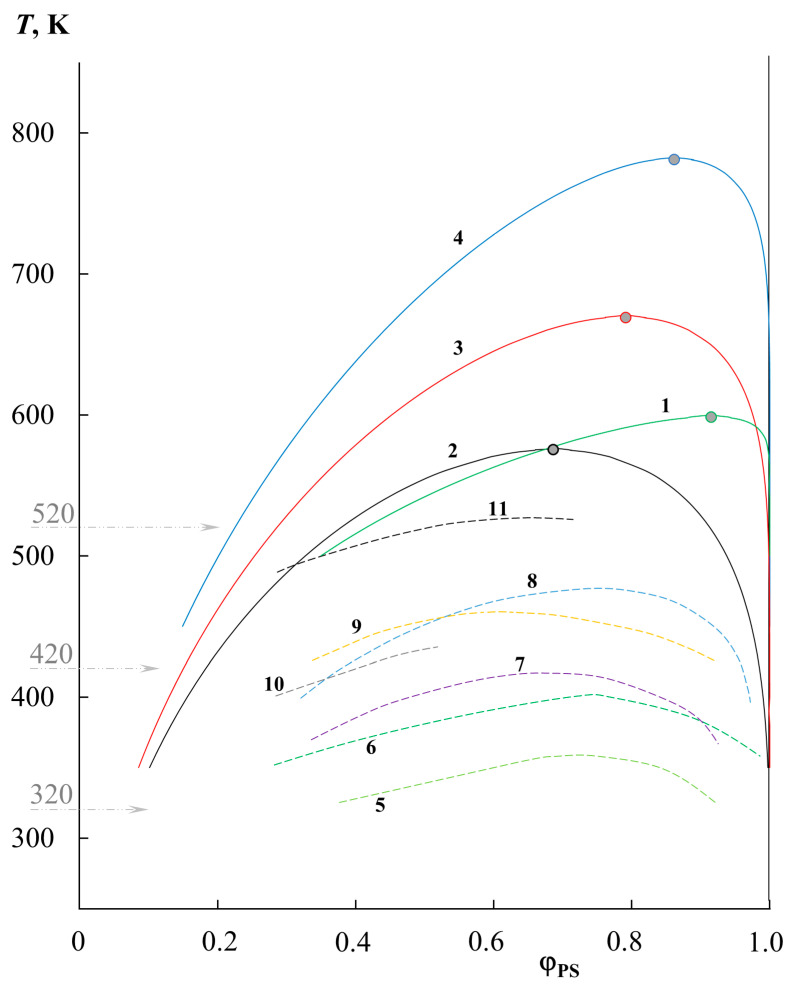
Generalized data on phase equilibrium in the PS–PMMA system of various molecular weights: 1—PS0.8k–PMMA89k, 2—PS2.4k–PMMA11k, 3—PS2.4k–PMMA33k, 4—PS2.4k–PMMA89k, 5—PS0.8k–PMMA6.35k [11], 6—PS0.8k–PMMA12.9k [11], 7—PS1.39k–PMMA6.35k [12], 8—PS1.39k–PMMA12k [12], 9—PS2.5k–PMMA6k [13], 10—PS2.95k–PMMA4.25k [14], 11—PS2.95k–PMMA10.55k [14]. Binodal curves 1–4 correspond to our simulation data, and binodal curves 5–11 represent binodal curves from different authors.

Figure 5 contains a graphical representation of all the calculated binodal curves obtained by us (solid lines), as well as the literature data on solubility (dashed lines). The numbering of these curves is shown in Table 1. It can be seen that the literature data complement the temperature–concentration field of the phase diagram well, with data on the solubility of PS and PMMA across a wide range of molecular weights. It is interesting to note that the approach to assessing the solubility of polymers based on their solubility parameters [3,25] generally did not take into account the effect of molecular weight, and only the chemical nature of the components. Thus, its calculated value according to the van Krevelen’s method for PS was 9.4 cal^1/2^/cm^3/2^, and for PMMA 9.14 cal^1/2^/cm^3/2^, and, according to other calculation methods, the range for PS was 9.0–9.4 cal^1/2^/cm^3/2^, and for PMMA 8.9–9.15 cal^1/2^/cm^3/2^ [25]. This difference suggested good polymer affinity and excellent solubility, which is not entirely true in practice and is highly dependent on molecular weight. Figure 5 shows that with an increase in *M*_PS_ and *M*_PMMA_, the region of the two-phase state increases, the solubility of polymers in each other decreases, the UCST shifts to the region of high temperatures, and the position of the critical point shifts from the region of medium concentrations towards a concentrated mixture with a strong divergence of the molecular weights of the components. At the same time, a full correlation is maintained between systems characterized by the same molecular weight of one of the components. For example, curves 1, 10, and 11 correspond to PS0.8k and the change in *M*_PMMA_ from 89k to 12.9k and 6.35k, respectively. A similar example can be given for *M*_PS_ 2.4–2.5k; these are curves 9, 2, 3, and 4, where the *M*_PMMA_ is 6k, 11k, 33k, and 89k, respectively.

**Figure 6:**
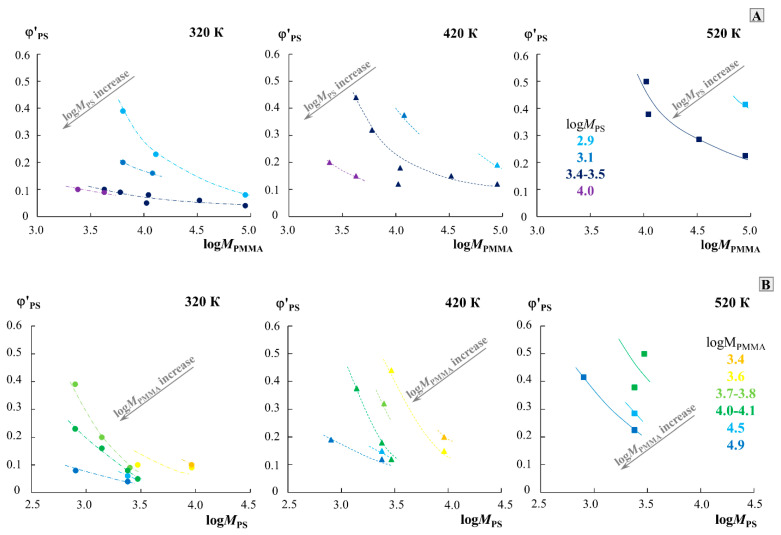
Dependence of the solubility of components in the PMMA phase on the molecular weight of PMMA (**A**) and PS (**B**) for isotherms of 320 K, 420 K, and 520 K. Different colors show data for one molecular weight of the second component. The arrow in each part of the figure indicates the direction of the dependence shift with an increase in the molecular weight of another component.

For a qualitative assessment of the effect of the molecular weight of the components, the data of the phase diagram were rebuilt along the cross section of three isotherms (320, 420 and 520 K) in the $\varphi_{\mathrm{PS}}^{\prime }-\log M_{\mathrm{PMMA}}$ coordinates for different $M_{\mathrm{PS}}$ and the $\varphi_{\mathrm{PS}}^{\prime }-\log M_{\mathrm{PS}}$ coordinates for different $M_{\mathrm{PMMA}}$ (Figure 6). To build these dependencies, only the data on the solubility of the left branch of the diagram (in the PMMA matrix) were used, since the right side of the diagram shows extremely low solubility for different systems at different temperatures. Dotted lines, without additional mathematical calculations, combine points of the same molecular weight value of the second component and show a trend in the solubility of the components. The arrow indicates the shift of these curves in the transition from the data group for one molecular weight of the second component to the data group on the other. It is clearly seen that the change in *M*_PS_ affects the solubility of these polymers to a greater extent than the change in *M*_PMMA_. For example, at 320 K, the $\log M_{\mathrm{PMMA}}$ of order 4 $\varphi_{\mathrm{PS}}^{\prime }$ changes from 0.23 to 0.07 with an increase in $\log M_{\mathrm{PS}}$ from 2.9 to 3.5. In this case, even for $\log M_{\mathrm{PS}}$ of the order of 3.5, $\varphi_{\mathrm{PS}}^{\prime }$ changes only from 0.1 to 0.04 as $\log M_{\mathrm{PMMA}}$ increases from 3.4 to 4.9. When passing from 320 to 420 K, and then to 520 K, the solubility curves shift from left to right in the presented coordinates, which is associated with an increase in the solubility of the components and the formation of completely homogeneous solutions of a number of mixtures. This dependence can serve as a nomogram for estimating and predicting the solubility in the PS–PMMA system of various ratios of the molecular weights of PS and PMMA at different temperatures.

The obtained data do not show a good correlation with the calculated values of the limiting solubility of PS at room temperature [26], calculated from ternary mixtures in a common solvent based on the affinity of polymers and solvent, and the effect of this affinity on the total solubility. Thus, for PMMA87k, it was expected that the limiting solubility of PS in it would vary from 0.9 to 44.5% with a change in $M_{\mathrm{PS}}$ from 370 to 2.66 kDa.

**Figure 7:**
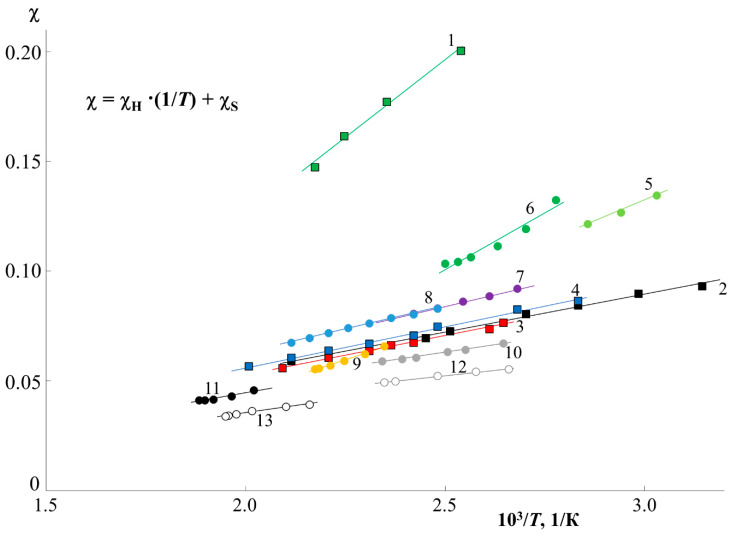
Generalized data on the temperature dependence of χ in the PS–PMMA system of various molecular weights. Curve designations correspond to Figure 5 and Table 1.

**Figure 8:**
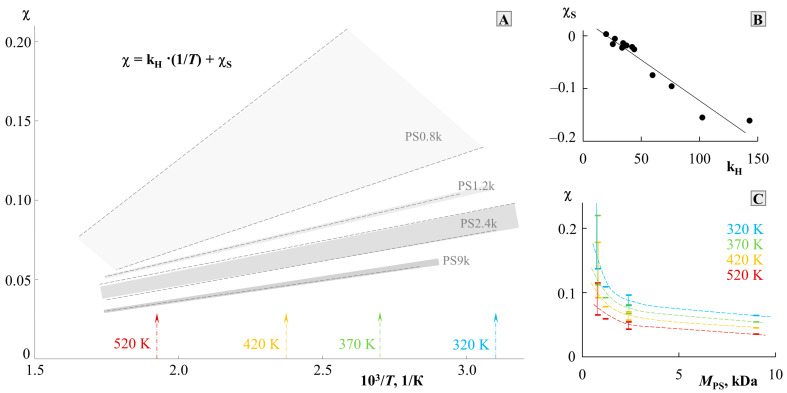
Analysis of the temperature dependence of χ (**A**) with a cross section along isotherms (**C**) and the correlation between χ_S_ and k_H_ (**B**). See details in the text.

To assess the thermodynamics of mixing PS and PMMA, we constructed a generalized temperature dependence of the interaction parameter in accordance with the data for all systems (Figure 7). It can be seen that all dependences are straightforward and can be described by an equation of the χ=kH·1T+χS type, where kH·1T=χH and is the enthalpy contribution to the Flory–Huggins parameter, and $\chi_{S}$ is the entropy contribution [3,23,27]. The numerical values of $k_{H}$ and $\chi_{S}$ are presented in Table 1. The correlation between $k_{H}$ and $\chi_{S}$ is shown in Figure 8B. It is linear, as was noted earlier for many other systems [23], which corresponds to a change in the predominance of the contribution of the enthalpy and entropy components during the transition of the system from one *M*_w_ to another. In fact, $k_{H}$ corresponds to the tangent of a slope of such dependence, and, according to Figure 7, changes little for a larger number of systems. PS0.8k behaves well, for which the angle of inclination of the straight lines sharply increases. It can be seen that for PS0.8k, the change in $M_{\mathrm{PMMA}}$ has a stronger effect on the position of the straight line than for other $M_{\mathrm{PS}}$. Within the previously indicated error in determining the solubility parameters, linear interpolation can fluctuate in a certain range of values. This will affect the spread of $\chi_{S}$ and $k_{H}$ data. We noted that for successful simulation of phase diagrams and convergence of binodal curves at the critical point, the average values of the approximating straight lines (presented in Table 1) are best suited. We used them for subsequent calculations. To assess the influence of molecular weight, the dependence χ−1T was rebuilt in the form of conditional zones (Figure 8A). To perform this, all systems were divided into groups with a common $M_{\mathrm{PS}}$, and the boundaries of each conditional zone were the extreme temperature dependences of the interaction parameter of this group. The molecular weight of the PS of each group is indicated on the schematic diagram. So, the zone for PS0.8k is much wider than for the rest. And, if for PS1.2k and PS9k this could be attributed to insufficient data, then the sample of systems with PS2.4k is quite representative. Rebuilding this dependence over several isothermal sections, we obtained a schematic dependence of χ on $M_{\mathrm{PS}}$ (Figure 8C). In this case, a range of potential values was used for each point. It can be seen that such dependences are almost linear for $M_{\mathrm{PS}}$ from 1 to 10 kDa. And, only when $M_{\mathrm{PS}}$ approaches 0.8 kDa, a sharp increase in the χ values is observed. Moreover, if we evaluate the lower limit of values in this region, then χ fits well with the continuation of the linear dependence. It turns out that only the most oligomeric (*M* < 1 kDa) representatives of the homologous series have an excellent effect on the mixing of polymers. We believe that this may be due to the increasing influence of end groups for such low-molecular-weight objects, similarly to what can be seen in the glass transition temperature of PS with a sharp decrease in its molecular weight down to 0.8 kDa [25].

**Figure 9:**
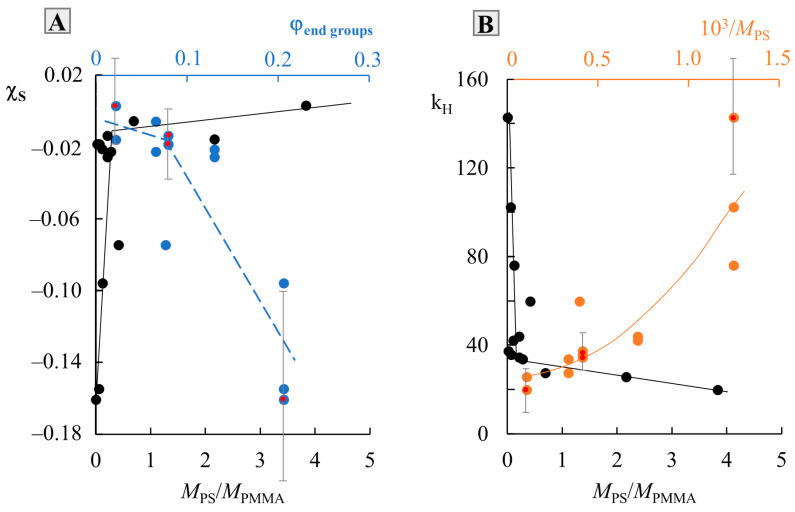
Dependence of χ_S_ (**A**) and k_H_ (**B**) on PS molecular weight. See details in the text.

Previously, work has already been carried out to assess the effect of end groups on the Flory–Huggins interaction parameter, not only in polymer–polymer systems, but also in more complex polymer–copolymer systems [28]. The presence of end groups at a short length of the macromolecular chain (oligomers) can introduce more disorder into the conformation of the polymer, and, consequently, affect its thermodynamics when mixed with another polymer. The entropy parameter $\chi_{S}$ just estimates the measure of order–disorder in the system. To quantify the effect of end groups on the entropy component of the interaction parameter, a dependence was constructed in the coordinates $\chi_{S}-\varphi_{\mathrm{end}\;\mathrm{groups}}$ (Figure 9, blue curve), where $\varphi_{\mathrm{end}\;\mathrm{groups}}$ is the proportion of end groups, calculated from the assumption that each macromolecular chain has two groups. Then, the proportion of end groups is the ratio of the number of end groups to the number of repeating units in accordance with the degree of polymerization, r. It can be seen that such a dependence has a broken character and allows one to conditionally estimate the values of $\chi_{S}$ of the order of −0.02 and $\varphi_{\mathrm{end}\;\mathrm{groups}}$ of the order of 0.08, up to which the influence of the end groups has almost no effect on the conformational stacking of macromolecular chains. After that, even a slight increase in the proportion of end groups leads to a sharp change in $\chi_{S}$, and, consequently, to a conformational rearrangement of the entire mixture. It is especially worth noting that such changes in $\chi_{S}$ occur symbatically with changes in the ratio MPSMPMMA (black broken curves in Figure 9). It can be seen that a sharp increase in $\chi_{S}$ and a decrease in $k_{H}$ are replaced by a weak effect with an increase in MPSMPMMA. The numerical value of the break area corresponds to MPSMPMMA=0.22 for both $\chi_{S}$ and $k_{H}$. The orange curve shows the dependence of $k_{H}$ on the inverse value of $M_{\mathrm{PS}}$ and in fact is directly proportional to the increase in the proportion of end groups. Additionally, red dots are marked in Figure 9, which correspond to the systems we studied and one system of the authors. For these points, the range of fluctuations in the values of $\chi_{S}$ and $k_{H}$ was calculated in the case of maximum deviations in one direction and the other in the approximating temperature dependence of the interaction parameter within the known calculated error. It can be seen that the trend towards a broken or curvilinear character of the change in the values of $\chi_{S}$ and $k_{H}$ from the content of end groups or the reciprocal of the $M_{\mathrm{PS}}$ remains.

**Figure 10:**
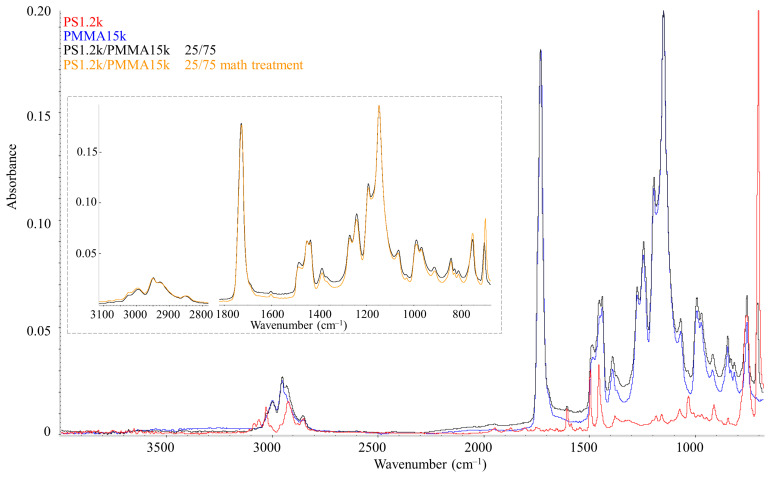
IR spectra of PS1.2k (red spectrum), PMMA15k (blue spectrum), and their mixtures in a ratio of 25/75 (black spectrum). The inset compares the spectrum of the mixture (black) with the model spectrum of the mixture (orange).

Figure 10 shows IR spectroscopy data for PS1.2k (red spectrum) and PMMA15k (blue spectrum). These spectra are characterized by standard absorption bands for these polymers [29,30]. In the range of 3100–2800 cm^–1^, there are stretching vibrations between carbon and hydrogen, and, unlike PMMA, PS has vibrations near the double bonds of the benzene ring (3100–3000 cm^−1^). The most intense absorption peaks of PMMA correspond to the C=O carbonyl group (1729 cm^−1^) and C–O stretching vibrations (1200–1100 cm^−1^). For PS, the most recognizable are the bands of double bonds of the benzene ring in the region of 1900–1600 cm^−1^ and the band of bending vibrations of this ring at 699 cm^–1^. To study the IR spectrum of the mixture, such a PS–PMMA composition was chosen, which corresponded to a homogeneous region in the phase diagram at 400 K. The spectrum of the PS1.2k/PMMA15k blend in the ratio 25/75 (black) repeats the spectra of the initial polymers in terms of the position of the absorption bands. For a quantitative analysis of the mixture spectrum, the spectra of the initial polymers were subjected to special mathematical treatment—the mathematical addition of the spectra in a given proportion. Such an addition spectrum is also shown in Figure 10 (orange spectrum inset). It is clearly seen that it is in full correlation with the experimentally obtained IR spectrum of the mixture (black), including in the range of 1125–1060 cm^−1^, which according to [29,31] is especially sensitive to conformational rearrangements of PMMA. This indicates the absence of any interactions between the functional groups of PS and PMMA upon mixing; additionally, the benzene ring and the carbonyl group are inert to each other.

## 3. Experimental

Monodisperse PS (Aldrich, St. Louis, Missouri, USA) with an average molecular weight *M*_w_ = 0.8 kDa (*M*_n_ = 0.74 kDa, *M*_w_/*M*_n_ =1.11, density 1.05 g/cm^3^, designated as PS0.8k), and *M*_w_ = 2.4 kDa (*M*_n_ = 2.17 kDa, *M*_w_/*M*_n_ =1.07, density 1.05 g/cm^3^, PS2.4k), monodisperse PMMA (Fluka, Germany, and Acros, Belgium) with *M*_w_ = 2 kDa (*M*_n_ = 1.74 kDa, *M*_w_/*M*_n_ =1.15, density 1.2 g/cm^3^, PMMA2k), *M*_w_ =11 kDa (*M*_n_ = 10.4 kDa, *M*_w_/*M*_n_ =1.06, density 1.2 g/cm^3^, PMMA11k), *M*_w_ = 33 kDa (density 1.18 g/cm^3^, PMMA33k), and *M*_w_ = 89 kDa (PMMA89k) were used as objects of study.

The solubility of polymers in each other was determined using optical interferometry [32] on an ODA-2 diffusiometer (IPCE RAS, Moscow, Russia). All measurements were made on pressed polymer films, which were 100–120 µm thick. Films 5 × 10 mm in size were placed in a temperature-controlled diffusion cell between two optically transparent glasses. A translucent layer of metal (Ni-Cr alloy) was deposited on the inner sides of the glasses by means of the thermal vacuum deposition method in VUP-4 (Russia). The wedge-shaped gap between the glasses was formed using special metal retainers of different thicknesses, 80 and 100 µm, and made an angle of ~2 degrees. The cell glasses were brought into optical contact with the polymers at a temperature slightly above the glass transition (melting) temperature of the polymers. The light source was a helium–neon laser with a wavelength of 632 nm. Using a digital camera through a microscope, we observed and recorded the interference of light passing perpendicular to the end surface of the contacting films. From the interferograms, the emerging concentration profile of the interdiffusion of polymers in the area of their contact was calculated. The temperature range of the study was from 370 to 500 K in the stepwise heating mode and from 500 to 300 K in the slow cooling mode. The step on the temperature scale was 10–20 K, with minimum temperature control for 30 min. The technique for conducting the experiment and processing interferograms did not differ from the traditional one [21,22]. The solubility of polymers was judged from the composition of saturated solutions that were established at the interface in the process of interdiffusion of the components. Fragments of binodal curves were constructed from the temperature dependences of the compositions of coexisting phases. The equilibrium of boundary curves (binodals) was proven by determining the reproducibility of the compositions of coexisting phases, measured in stepwise “heating–cooling” modes.

A Nicolet iN10 ATR-FTIR spectrometer (Thermo Scientific, Waltham, MA, USA) was used to analyze the character of intermolecular interactions of PS1.2k, PMMA15k components, and their mixture. Studies were carried out in the spectral region 675–4000 cm^−1^ on a germanium crystal in the ATR mode by accumulating 128 scans with a resolution of 4 cm^−1^ at room temperature. Polymer films and their mixture were obtained by pressing at a temperature of 400 K. IR spectra were processed by means of transformation into the absorbance mode with automatic baseline correction using the Omnic 9 software (Thermo Scientific, Waltham, MA, USA).

## 4. Conclusions

Optical interferometry was used to obtain data on the solubility of oligomeric PS and PMMA of various molecular weights from 2 to 89 kDa. It has been shown that the PS0.8k–PMMA2k system is completely mutually soluble in the temperature range from 300 to 500 K. Systems with other molecular weights are characterized by limited solubility regions of no more than 20% PS in PMMA and 10% PMMA in PS up to 450 K. The more M_PMMA_, the lower the solubility of PS in PMMA, and thus the phase diagram is more and more asymmetric. According to the previously proposed approach for the simulation of binodal and spinodal curves, within the framework of the Flory–Huggins theory, the interaction parameters of all systems were calculated, their temperature dependences were plotted, and they were extrapolated to the calculated values of the critical point. The constructed phase diagrams are characterized by the presence of UCST and contain complete information about the homogeneous and heterogeneous regions of all the studied systems. A comparative analysis with the data of other authors on the solubility in the PS–PMMA systems of other molecular weights was carried out. There is an excellent correlation of results. It should be noted that the approach we proposed for predicting phase diagrams shows a better result than in other works. For a qualitative assessment of the effect of the molecular weight of the components, the data of the phase diagram were reconstructed over the cross section of three isotherms of 320, 420, and 520 K. It was shown that the solubility of PS in PMMA noticeably decreases with an increasing molecular weight, with *M*_PS_ affected to a greater extent than *M*_PMMA_. The obtained dependences can serve as nomograms for predicting the solubility of various ratios of PS and PMMA molecular weights.

The thermodynamics of component mixing were estimated from the generalized temperature dependence of the interaction parameter constructed for all systems. It is shown that the enthalpy and entropy components are linearly dependent on each other and a decrease in the contribution of one leads to an increase in the contribution of the other. The dependence of the interaction parameter on the molecular weight for different isothermal cross sections shows a linear dependence with decreasing *M*_PS_ from 9 to 1 kDa, and a sharp increase upon going to PS0.8k. We believe that this is due to the growing influence of end groups for such oligomeric components. The IR spectroscopy of the initial polymers and their mixture showed that there were no interactions between the functional groups of PS and PMMA.

## Figures and Tables

**Table 1:** Calculated data on the parameters of the critical point and the temperature dependence of the interaction parameter for PS-PMMA systems with different molecular weights.

N	System	χ_cr_	UCST, K	φ_(PS)cr_	$\mathrm{Equation}\;\chi =A+B\cdot \frac{1}{T}$	R^2^
1	PS0.8k–PMMA89k	0.0776	599	0.915	χ = 142.8·(1/*T*) − 0.161	0.993
2	PS2.4k–PMMA11k	0.0461	575	0.686	χ = 34.4·(1/*T*) − 0.0137	0.995
3	PS2.4k–PMMA33k	0.0346	669	0.791	χ = 35.7·(1/*T*) − 0.0186	0.994
4	PS2.4k–PMMA89k	0.0292	781	0.861	χ = 37.2·(1/*T*) − 0.0184	0.995
5	PS0.8k–PMMA6.35k [11]	0.119	353	0.743	χ = 75.9·(1/*T*) − 0.0959	0.991
6	PS0.8k–PMMA12.9k [11]	0.101	400	0.804	χ = 102.3·(1/*T*) − 0.155	0.949
7	PS1.39k–PMMA6.35k [12]	0.0796	417	0.686	χ = 43.8·(1/*T*) − 0.0256	0.999
8	PS1.39k–PMMA12k [12]	0.0665	476	0.750	χ = 42.1·(1/*T*) − 0.0213	0.996
9	PS2.5k–PMMA6k [13]	0.0553	461	0.612	χ = 59.6·(1/*T*) − 0.0746	0.993
10	PS2.95k–PMMA4.25k [14]	0.0582	433	0.550	χ = 27.4·(1/*T*) − 0.0056	0.993
11	PS2.95k–PMMA10.55k [14]	0.0406	526	0.659	χ = 33.6·(1/*T*) − 0.0226	0.955
12	PS9.2k–PMMA2.4k [14]	0.0482	427	0.342	χ = 19.7·(1/*T*) + 0.003	0.994
13	PS9.2k–PMMA4.25k [14]	0.0337	524	0.409	χ = 25.6·(1/*T*) − 0.0158	0.986

## Data Availability

The data presented in this study are available on request from the corresponding author.

## Supplementary Materials

The following supporting information can be downloaded at: https://www.mdpi.com/article/10.3390/molecules28135259/s1, Figure S1 Method for simulation phase diagrams from fragments of binodal curves in the framework of the Flory-Huggins theory.
