# Supplementary material for: Effect of Molecular Weight on Phase Equilibrium in the Polystyrene–Poly(methyl methacrylate) System
Source: Molecules. 2023 Jul 7;28(13):5259. doi: 10.3390/molecules28135259 (PMC10343856; doi:10.3390/molecules28135259)
Supplement: Supplementary file 1 [file molecules-28-05259-s001.zip › molecules-2454407-supplementary.pdf]

Supplementary Materials

THEORETICAL METHODOLOGY [24]

Traditionally, in the framework of the Flory-Huggins theory, the free energy of mixing of two polymers is represented as follows:

$$\Delta G = RT\frac{V}{V_r}\left[\frac{\varphi_1}{r_1}ln\varphi_1 + \frac{\varphi_2}{r_2}ln\varphi_2 + \chi_{12}\varphi_1\varphi_2\right] \tag{1}$$

where R is the universal gas constant, T is the temperature, V is the polymer mixing volume, V_r is the reference volume, usually taken as the molar volume of a repeating unit in the system (in calculations, a reference volume of 100 cm^3/mol is traditionally used [3]), φ_1 and φ_2 are the volume fractions of the first and second components in the system, r_1 and r_2 are their polymerization degrees, χ_{12} is the Flory-Huggins interaction parameter.

Expressions for chemical potentials are as follows:

$$\begin{aligned}\Delta\mu_1 &= RT\left[\frac{ln\varphi_1}{r_1} + \left(\frac{1}{r_1} - \frac{1}{r_2}\right)\varphi_2 + \chi_{12}\varphi_2^2\right]\\ \Delta\mu_2 &= RT\left[\frac{ln\varphi_2}{r_2} + \left(\frac{1}{r_2} - \frac{1}{r_1}\right)\varphi_1 + \chi_{21}\varphi_1^2\right]\end{aligned} \tag{2}$$

The conditions at the critical point are as follows:

$$\varphi_{1,cr} = \frac{\sqrt{r_2}}{\sqrt{r_1} + \sqrt{r_2}} \qquad \varphi_{2,cr} = \frac{\sqrt{r_1}}{\sqrt{r_1} + \sqrt{r_2}} \tag{3}$$

$$\chi_{cr} = \frac{1}{2}\left(\frac{1}{\sqrt{r_1}} + \frac{1}{\sqrt{r_2}}\right)^2 \tag{4}$$

Spinodal concentrations are defined as the points at which the second derivative of the free energy of mixing with respect to concentration is zero. The spinodal equation takes the form:

$$\frac{1}{r_1\varphi_{1,s}} + \frac{1}{r_2\varphi_{2,s}} - 2\chi = 0 \tag{5}$$

where χ is the averaged value of the pair interaction parameter.

The binodal equations are calculated from the condition that the chemical potentials of both polymers are equal in two coexisting phases:

$$\begin{aligned}\frac{ln(\varphi_1')}{r_1} + \left(\frac{1}{r_1} - \frac{1}{r_2}\right)\varphi_2' + \chi_{12}(\varphi_2')^2 &= \frac{ln(\varphi_1'')}{r_1} + \left(\frac{1}{r_1} - \frac{1}{r_2}\right)\varphi_2'' + \chi_{12}(\varphi_2'')^2\\ \frac{ln(\varphi_2')}{r_2} + \left(\frac{1}{r_2} - \frac{1}{r_1}\right)\varphi_1' + \chi_{21}(\varphi_1')^2 &= \frac{ln(\varphi_2'')}{r_2} + \left(\frac{1}{r_2} - \frac{1}{r_1}\right)\varphi_1'' + \chi_{21}(\varphi_1'')^2\end{aligned} \tag{6}$$

where $'$ and $''$ refer to different coexisting phases.

For thermodynamic analysis of the obtained experimental data on binodal curves (or their fragments), the presented equations lead to the following expressions for the pair interaction parameter:

$$\chi_{12} = \frac{ln(\varphi_1'') - ln(\varphi_1')}{r_1((\varphi_2')^2 - (\varphi_2'')^2)} - \left(\frac{1}{r_1} - \frac{1}{r_2}\right)\frac{1}{\varphi_2' + \varphi_2''} \quad (7)$$

$$\chi_{21} = \frac{ln(\varphi_2'') - ln(\varphi_2')}{r_2((\varphi_1')^2 - (\varphi_1'')^2)} - \left(\frac{1}{r_2} - \frac{1}{r_1}\right)\frac{1}{\varphi_1' + \varphi_1''} \quad (8)$$

The averaged value of the pair interaction parameter, assuming the absence of its concentration dependence, can be represented as follows:

$$\chi = \frac{\frac{ln(\varphi_1''/\varphi_1')}{r_1} - \frac{ln(\varphi_2''/\varphi_2')}{r_2}}{2(\varphi_2' - \varphi_2'')} \quad (9)$$

Figure below shows a scheme of the methodology for the thermodynamic analysis of binary polymer systems and the construction of generalized phase diagrams. At the initial stage, fragments of binodal curves are plotted based on the experimentally obtained data on the solubility of polymers in each other (**a**). Then, according to the compositions of the coexisting phases φ_1' and φ_1'' at different temperatures T, using the Equation (9), the numerical values of the pair interaction parameter χ are calculated and its temperature dependence is plotted (**b**). Extrapolation of this dependence to χ_{cr} makes it possible to obtain information about the critical solution temperature of the components (in this case, the upper critical solution temperature – UCST). In coordinates $\chi = A + B \cdot {}^{1}/_{T}$ it is possible to identify χ data in a wide range of temperatures at $T \geq T_{ex}$, $T \leq T_{ex}$, $T \geq$USCT. Based on these data, using Equations (5) and (6), the boundary spinodal (dashed line) and binodal (solid line) curves are calculated and a generalized phase diagram is constructed (**c**) in the selected temperature range, which traditionally denotes the region of homogeneous states (I), the region of labile structures (II), and the region of metastable states (III).

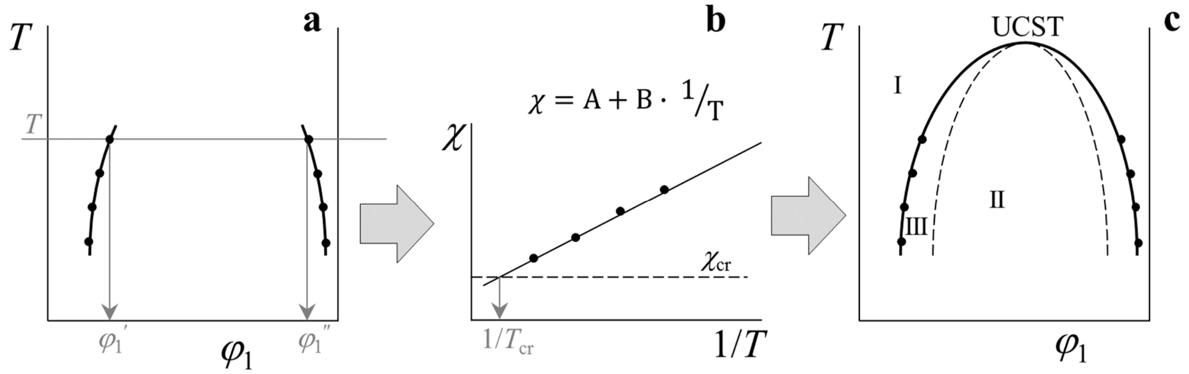

Figure S1 Method for simulation phase diagrams from fragments of binodal curves in the framework of the Flory-Huggins theory
